# RhANP attenuates endotoxin-derived cognitive dysfunction through subdiaphragmatic vagus nerve-mediated gut microbiota–brain axis

**DOI:** 10.1186/s12974-021-02356-z

**Published:** 2021-12-23

**Authors:** Yuming Wu, Yujing Zhang, Bing Xie, Amro Abdelgawad, Xiaoyan Chen, Mengqi Han, You Shang, Shiying Yuan, Jiancheng Zhang

**Affiliations:** 1 Department of Critical Care Medicine, Union Hospital, Tongji Medical College, Huazhong University of Science and Technology, 1277 Jiefang Avenue, 430022 Wuhan, People’s Republic of China; 2 Institute of Anesthesia and Critical Care Medicine, Union Hospital, Tongji Medical College, Huazhong University of Science and Technology, Wuhan, 430022 China; 3 Midyorks NHS Trust, Wakefield, England

**Keywords:** Cognition, Gut microbiota, LPS, Neuroinflammation, Subdiaphragmatic vagus nerve

## Abstract

**Background:**

Atrial natriuretic peptide (ANP) secreted from atrial myocytes is shown to possess anti-inflammatory, anti-oxidant and immunomodulatory effects. The aim of this study is to assess the effect of ANP on bacterial lipopolysaccharide (LPS)-induced endotoxemia-derived neuroinflammation and cognitive impairment.

**Methods:**

LPS (5 mg/kg) was given intraperitoneally to mice. Recombinant human ANP (rhANP) (1.0 mg/kg) was injected intravenously 24 h before and/or 10 min after LPS injection. Subdiaphragmatic vagotomy (SDV) was performed 14 days before LPS injection or 28 days before fecal microbiota transplantation (FMT). ANA-12 (0.5 mg/kg) was administrated intraperitoneally 30 min prior to rhANP treatment.

**Results:**

LPS (5.0 mg/kg) induced remarkable splenomegaly and an increase in the plasma cytokines at 24 h after LPS injection. There were positive correlations between spleen weight and plasma cytokines levels. LPS also led to increased protein levels of ionized calcium-binding adaptor molecule (iba)-1, cytokines and inducible nitric oxide synthase (iNOS) in the hippocampus. LPS impaired the natural and learned behavior, as demonstrated by an increase in the latency to eat the food in the buried food test and a decrease in the number of entries and duration in the novel arm in the Y maze test. Combined prophylactic and therapeutic treatment with rhANP reversed LPS-induced splenomegaly, hippocampal and peripheral inflammation as well as cognitive impairment. However, rhANP could not further enhance the protective effects of SDV on hippocampal and peripheral inflammation. We further found that PGF mice transplanted with fecal bacteria from rhANP-treated endotoxemia mice alleviated the decreased protein levels of hippocampal polyclonal phosphorylated tyrosine kinase receptor B (p-TrkB), brain-derived neurotrophic factor (BDNF) and cognitive impairment, which was abolished by SDV. Moreover, TrkB/BDNF signaling inhibitor ANA-12 abolished the improving effects of rhANP on LPS-induced cognitive impairment.

**Conclusions:**

Our results suggest that rhANP could mitigate LPS-induced hippocampal inflammation and cognitive dysfunction through subdiaphragmatic vagus nerve-mediated gut microbiota–brain axis.

**Supplementary Information:**

The online version contains supplementary material available at 10.1186/s12974-021-02356-z.

## Introduction

Studies of both animals and humans have shown a close relationship between systemic inflammation and neuropsychiatric disorders [1–3]. Systemic lpopolysaccharides (LPS) injection induces increase in the serum cytokines and alarmins could directly disrupt the blood–brain barrier (BBB) and alter BBB function, thus allowing proinflammatory immune cells and molecules to the central nervous system (CNS) [4–7]. LPS-challenged animal model to mimic Gram-negative bacterial infection is widely used to study the psychiatric and cognitive consequences of infection and systemic inflammation [8, 9]. It has been shown that systemic LPS-triggered neuroinflammation plays a pivotal role in the pathophysiology of endotoxemia-derived cognitive impairment [4, 10–15]. The intraperitoneal LPS injection was chosen as an animal model of endotoxemia in our present study, because it has more clinical relevance than intracranial LPS injection. An effective and timely attenuation of neuroinflammatory response through pharmacological and molecular interventions could improve endotoxemia-derived cognitive impairment [10, 12].

Atrial natriuretic peptide (ANP), a cardiovascular hormone mainly secreted by the heart atria [16], plays obvious protective roles in a variety of diseases (ventricular hypertrophy, myocardial injury, hypertension, tumor, acute lung injury, cerebral ischemia/reperfusion injury, and sepsis, etc.) [17–27]. ANP has immunomodulatory capacity by regulating innate immunity and adaptive immunity, including increasing macrophage phagocytosis and reactive oxygen species release, enhancing natural killer (NK) cytotoxicity, promoting dendritic cell-mediated T cell polarization, stimulating the differentiation of naive CD4^+^ cells toward the T helper (Th) 2 and/or Th17 phenotype, inhibiting the expression of proinflammatory mediators and adhesion molecules [17, 28–31]. We previously reported a significantly positive correlation between the plasma ANP levels and early recovery of immune function in septic patients [32]. ANP is shown to be capable of suppressing inflammatory mediators LPS and tumor necrosis factor (TNF)-α-induced increase in endothelial cell (EC) permeability and preserving EC barrier function [18]. In an experimental model of systemic LPS challenge-induced sepsis, ANP pretreatment could improve survival [24]. Furthermore, a prospective randomized controlled, observer‑blinded study found that ANP treatment could alleviate intestinal injury and reduce the length of intensive care unit (ICU) stay and mortality rate in patients with septic shock [25]. However, there are currently no reports of the effects of ANP on systemic LPS-induced endotoxemia-derived neuroinflammation and cognitive impairment.

We have previously demonstrated an important role of subdiaphragmatic vagus nerve-mediated gut–brain axis in LPS-triggered endotoxemia or sepsis-induced neuropsychiatric disorder [33, 34]. Natriuretic peptide receptor A (NPR-A), a major receptor for ANP, is shown to be highly expressed in small intestinal epithelial cells [35]. Whether ANP could attenuate systemic LPS-triggered neuroinflammation and cognitive dysfunction through subdiaphragmatic vagus nerve-mediated gut–brain axis remains unknown. The aim of the present study was to evaluate the prophylactic and/or therapeutic effects of recombinant human ANP (rhANP) on systemic inflammation, neuroinflammation and delirium-related cognitive impairment produced by LPS (5 mg/kg)-induced endotoxemia.

## Materials and methods

### Animals

Adult male C57BL/6 J mice (22.0–25.0 g, 8–10 weeks) were purchased from Vital River Laboratory Animal Technology Co Ltd., Beijing, China. The mice were housed in a specific pathogen-free facility with a 12-h light and dark cycle (lights on, 6:00 AM–6:00 PM) at 22 ± 2 ℃ and were supplied with food and water ad libitum. All procedures were performed in accordance with the National Institutes of Health guide for the care and use of Laboratory animals (NIH Publications No. 8023, revised 1978). All animal experiments were approved by the committee of experimental animals of Tongji Medical College (Permission number: S2552).

### Treatment

Animals were randomized to each groups (*n* = 10/group). 0.9% saline (10 ml/kg), or LPS (5 mg/kg; L-4130, serotype 0111:B4; Sigma-Aldrich, St. Louis, MO, USA) was given intraperitoneally (i.p.) to mice. RhANP (1.0 mg/kg; GL Biochem Ltd., Shanghai, China) or 0.9% saline (10 ml/kg) was injected intravenously to mice in three different protocols as follows: (1) single dose administration 24 h prior to LPS injection; (2) single dose administration 10 min after LPS injection; and (3) Administration twice 24 h before and 10 min after LPS injection. ANA-12 (0.5 mg/kg; Maybridge, Cornwall, UK), was dissolved in PBS containing 17% dimethylsulfoxide (DMSO) [32] and administrated i.p. to mice 30 min prior to treatment with rhANP.

The mice were deeply anesthetized 24 h after injection of saline or LPS. Blood was collected via cardiac puncture, placed into tubes containing ethylenediaminetetraacetic acid (EDTA), and immediately centrifuged at 3000 *g* for 5 min at 4 ℃ to get plasma and then stored at − 80 ℃. The bilateral prefrontal cortex (PFC) and hippocampus were collected rapidly and stored at – 80 ℃. The weights of spleens were recorded immediately after spleen removal.

### Total subdiaphragmatic vagotomy (SDV)

Bilateral SDV or sham operation was performed 14 days prior to LPS injection or preparation for pseudo germ-free (PGF) modeling according to our previous studies [32, 36, 37]. Briefly, the abdomen was sterilized and a right transverse abdominal incision was made to expose the gastroesophageal junction keeping costal arc, liver, and stomach out of sight. The dorsal and ventral truncal branches of the vagus nerve were transected under a stereodissection microscope. The incision was closed by suture. Fourteen days after SDV, the observation of an increased stomach size and inspection of vagal nerve endings at sacrifice using microscopy indicated a successful vagotomy. For sham surgery, the vagus nerves were similarly exposed but not cut.

### PGF mice modeling

Based on our previous study [37], broad-spectrum antibiotics (ampicillin 1 g/L, neomycin sulfate 1 g/L, and metronidazole 1 g/L; Sigma-Aldrich Co. Ltd, USA) dissolved in drinking water were given ad libitum to mice for 14 consecutive days. The drinking solution was renewed every 2 days.

### Fecal microbiota transplantation (FMT)

FMT was conducted according to our previous studies [32, 37]. The fecal donor mice received treatment with rhANP or 0.9% saline twice 24 h before and 10 min after injection of LPS or 0.9% saline. 24 h after LPS injection, mice were placed in a clean cage with sterilized filter paper on the bottom. Fresh feces were collected into sterile cryotubes and immediately stored at − 80 ℃. 1 g of feces from donor mice was homogenized in 10 ml of sterile phosphate-buffered saline (PBS) and then suspended. At 24 h after PGF mice modeling was established, the fecal bacteria suspension was administrated to each PGF mouse recipient by intra-gastric gavage of 200 μl solution once a day for 14 consecutive days.

### Enzyme-linked immunosorbent assay (ELISA)

The plasma levels of interleukin (IL)-6 (#88-7064, Invitrogen, Camarillo, CA, USA), IL-17A (#88-7371, Invitrogen, Camarillo, CA, USA), interferon (IFN)-γ (#88-8314, Invitrogen, Camarillo, CA, USA), and TNF-α (#88-7324, Invitrogen, Camarillo, CA, USA) were measured using commercial ELISA kits according to the manufacturer’s instructions.

### Western blotting

Tissue samples from the PFC and hippocampus were homogenized in ice cold Laemmli lysis buffer, and then centrifuged at 3000 *g* for 10 min at 4 ℃ to get the supernatants. Equal amounts of protein were separated using 10% sodium dodecyl sulfate–polyacrylamide gel electrophoresis gels and transferred onto polyvinylidene difluoride membranes (Millipore, Temecula, CA, USA) using a Trans Blot Mini Cell (Bio-Rad). The membranes were blocked in 5% non-fat dried milk for 1 h at room temperature, and then incubated with the following primary antibodies: rabbit polyclonal anti-ionized calcium-binding adapter molecule 1 (iba-1; 1:1000, #016-20001: Wako Pure Chemical Industries, Ltd., Tokyo, Japan), rabbit monoclonal anti-IL-6 (1:1000, #12912S, Cell Signaling Technology, Inc., Danvers, MA, USA), rabbit monoclonal anti-IL-17A (1:1000, #A0688, ABclonal, Wuhan, China), rabbit monoclonal anti-IFN-γ (1:1000, #A12450, ABclonal, Wuhan, China), rabbit polyclonal anti-TNF-α (1:1000, #A0277, ABclonal, Wuhan, China), rabbit monoclonal anti-inducible nitric oxide synthase (iNOS; 1:1000, #ab3523, Abcam, Cambridge, MA, USA), brain-derived neurotrophic factor (BDNF; 1:1000, #A4873, ABclonal, Wuhan, China), rabbit polyclonal phosphorylated tyrosine kinase receptor B (p-TrkB; 1:1000, #AP0423, ABclonal, Wuhan, China), rabbit polyclonal total TrkB (t-TrkB; 1:1000, #A2099, ABclonal, Wuhan, China), and mouse monoclonal anti-β-actin (1:1000, #A2319, ABclonal, Wuhan, China) overnight at 4 ℃. The membranes were washed and incubated with horseradish peroxidase-conjugated anti-rabbit or anti-mouse secondary antibody (1:5000) for 1 h at room temperature. Chemiluminescence detection was carried out with ECL Western Blotting Detection Reagents (Beyotime, China) plus BioWest enhanced chemiluminescence (UVP, Upland, CA, USA). Band intensity was quantified with Image J software (National Institutes of Health, Bethesda, MD, USA).

### Statistical analysis

**Fig. 1:**
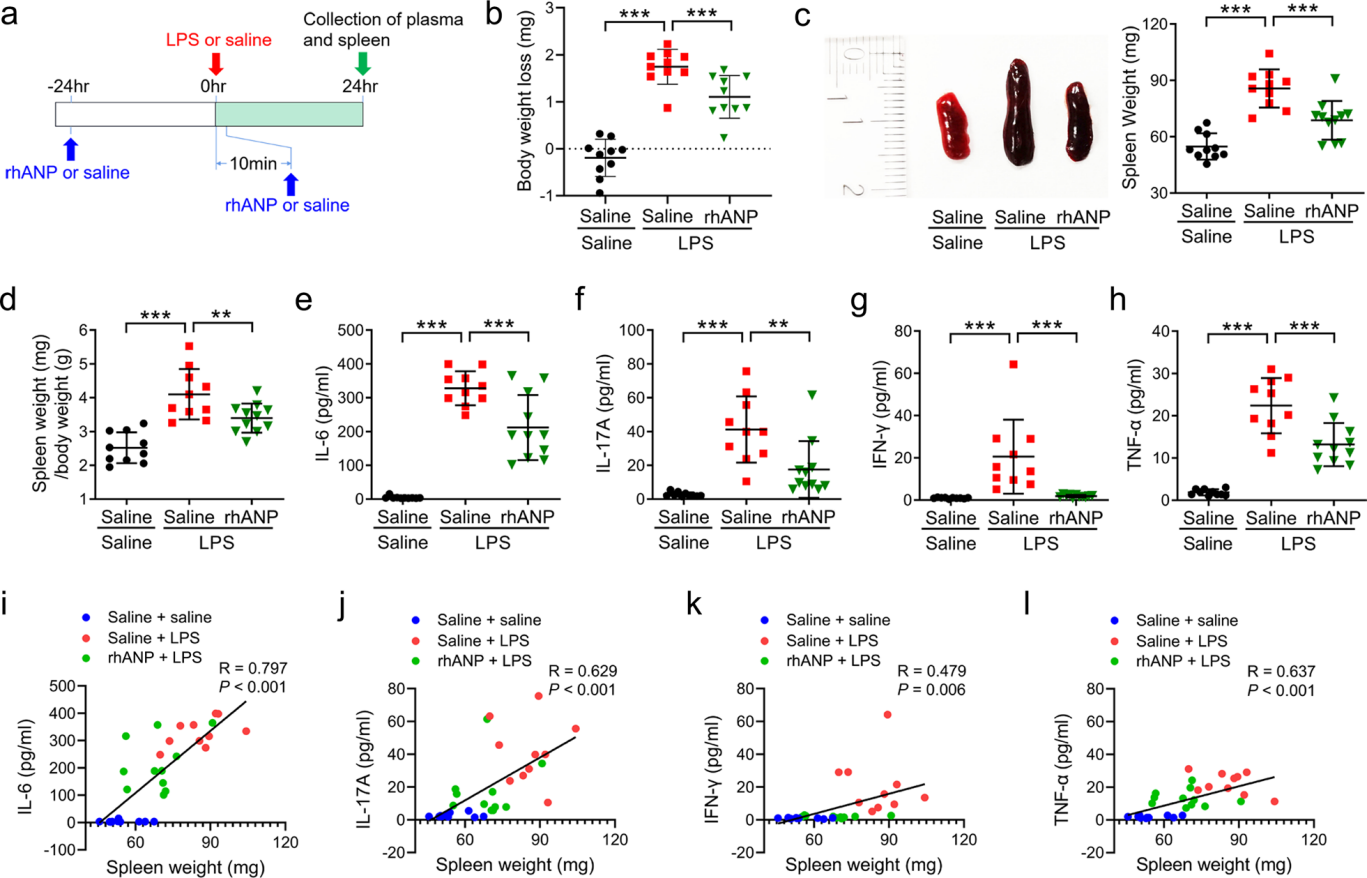
Effects of rhANP on plasma inflammatory cytokines after LPS-triggered endotoxemia.** a** Treatment schedule. Mice were intraperitoneally injected with lipopolysaccharides (LPS, 5 mg/kg) or 0.9% saline. Recombinant human ANP (rhANP; 1.0 mg/kg) or 0.9% saline were intraperitoneally injected to mice 24 h before and 10 min after LPS injection. Spleen and plasma were collected 24 h after injection of LPS or 0.9% saline. **b** Body weight loss in mice treated with rhANP or 0.9% saline 24 h after injection of LPS or 0.9% saline (one-way ANOVA: *F*_2,27_ = 58.21, *P* < 0.0001). **c** Representative picture of spleen and spleen weight (one-way ANOVA: *F*_2,28_ = 27.53, *P* < 0.0001). **d** Ratio of spleen weight/body weight (one-way ANOVA: *F*_2,28_ = 20.17, *P* < 0.0001). **e** Plasma levels of interleukin (IL)-6 (one-way ANOVA: *F*_2,28_ = 65.35, *P* < 0.0001). **f** Plasma levels of IL-17A (one-way ANOVA: *F*_2,28_ = 16.82, *P* < 0.0001). **g** Plasma levels of interferon (IFN)-γ (one-way ANOVA: *F*_2,28_ = 12.62, *P* < 0.0001). **h** Plasma levels of tumor necrosis factor (TNF)-α (one-way ANOVA: *F*_2,28_ = 51.43, *P* < 0.0001). **i** There was a positive correlation (r = 0.797, *P* < 0.001) between spleen weight and plasma IL-6. **j** There was a positive correlation (*r* = 0.629, *P* < 0.001) between spleen weight and plasma IL-17A. **k** There was a positive correlation (*r* = 0.479, *P* = 0.006) between spleen weight and plasma IFN-γ. **l** Positive correlation (*r* = 0.637, *P* < 0.001) between spleen weight and plasma TNF-α was observed. Data are shown as mean ± SEM, *n* = 10 or 11/group. ^**^P < 0.01, ^***^P < 0.0001

**Fig. 2:**
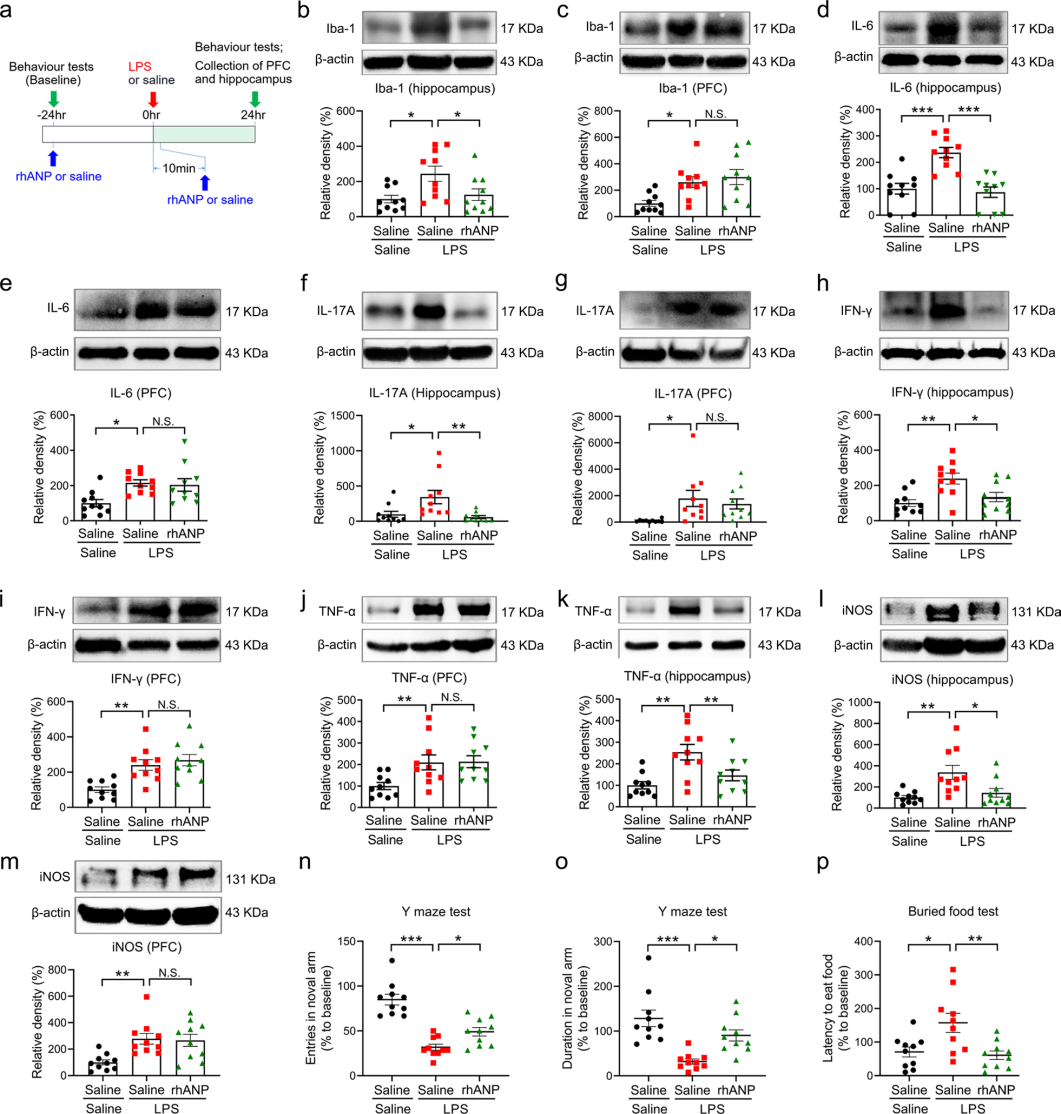
Effects of rhANP on the neuroinflammation and cognitive function after LPS-triggered endotoxemia. **a** Treatment schedule. Mice were intraperitoneally injected with lipopolysaccharides (LPS, 5 mg/kg) or 0.9% saline. Recombinant human ANP (rhANP; 1.0 mg/kg) or 0.9% saline were intraperitoneally injected to mice 24 h before and 10 min after LPS injection. The prefrontal cortex (PFC) and hippocampus were collected 24 h after injection of LPS or 0.9% saline. **b**, **c** Western blot analysis of ionized calcium-binding adapter molecule 1 (iba-1) in the prefrontal cortex (PFC) (one-way ANOVA: *F*_2,27_ = 6.170, *P* = 0.0062) and hippocampus (one-way ANOVA: *F*_2,27_ = 5.250, *P* = 0.0119). **d**, **e** Western blot analysis of interleukin (IL)-6 in the PFC (one-way ANOVA: *F*_2,27_ = 5.958, *P* = 0.0072) and hippocampus (one-way ANOVA: *F*_2,27_ = 17.60, *P* < 0.0001). **f**, **g** Western blot analysis of IL-17A in the PFC (one-way ANOVA: *F*_2,27_ = 4.498, *P* = 0.0206) and hippocampus (one-way ANOVA: *F*_2,27_ = 6.268, *P* = 0.0058). **h**, **i** Western blot analysis of interferon (IFN)-γ in the PFC (one-way ANOVA: *F*_2,27_ = 11.18, *P* = 0.0003) and hippocampus (one-way ANOVA: *F*_2,27_ = 7.611, *P* = 0.0024). **j**, **k** Western blot analysis of tumor necrosis factor (TNF)-α in the PFC (one-way ANOVA: *F*_2,27_ = 5.530, *P* = 0.0097) and hippocampus (one-way ANOVA: *F*_2,27_ = 8.550, *P* = 0.0013). **l**, **m** Western blot analysis of inducible nitric oxide synthase (iNOS) in the PFC (one-way ANOVA: *F*_2,27_ = 7.328, *P* = 0.0029) and hippocampus (one-way ANOVA: *F*_2,27_ = 7.597, *P* = 0.0024). **n**, **o** Entries in the novel arm (one-way ANOVA: *F*_2,27_ = 31.33, *P* < 0.0001) and duration in the novel arm (one-way ANOVA: *F*_2,27_ = 13.34, *P* < 0.0001) in the Y maze test. **p** Latency to eat food in the buried food test (one-way ANOVA: *F*_2,27_ = 7.129, *P* = 0.0033). Data are shown as mean ± SEM, *n* = 10/group. ^*^P < 0.05, ^**^P < 0.01, ^***^P < 0.0001; *N.S.* not significant

**Fig. 3:**
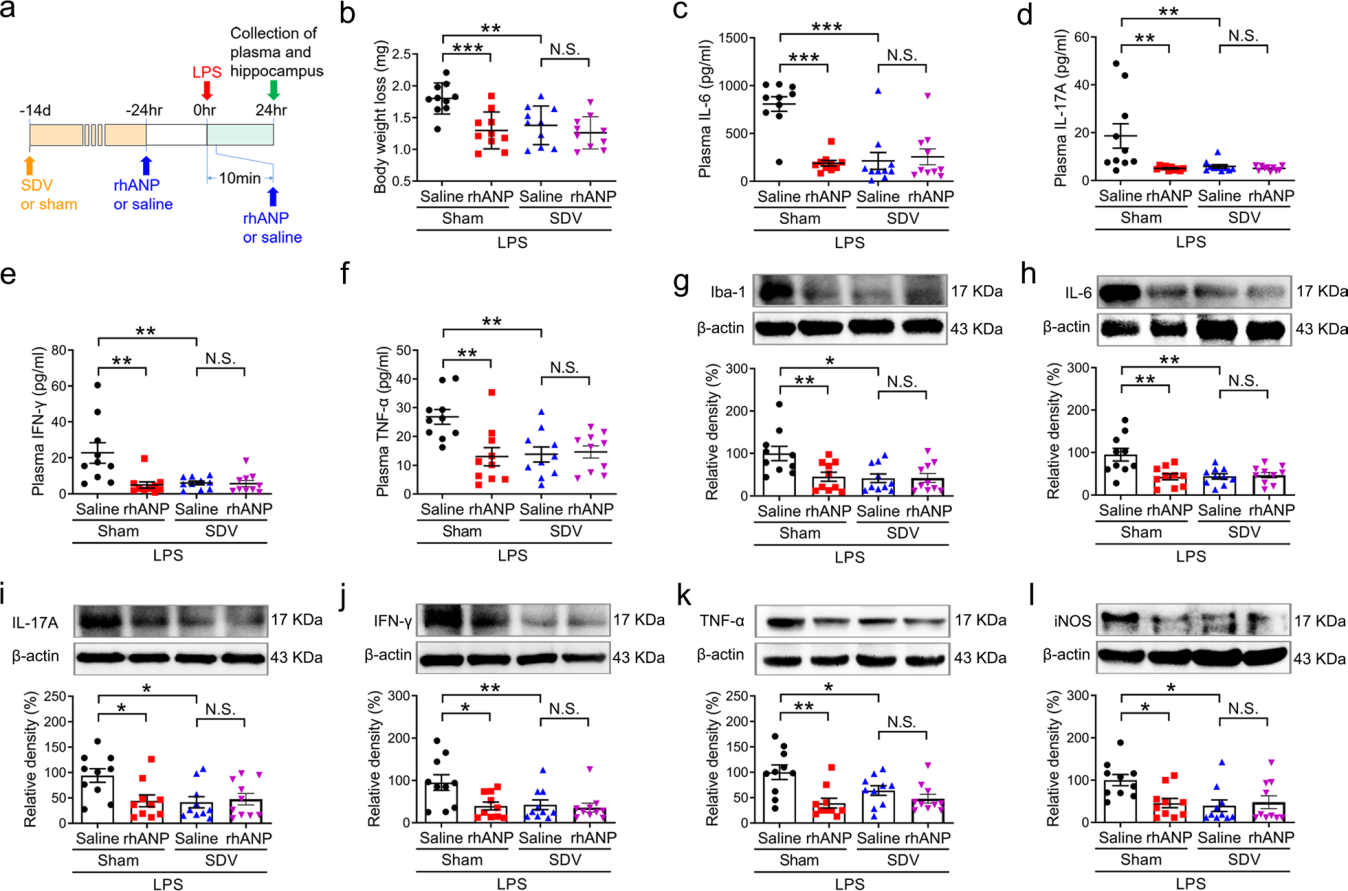
Roles of SDV in rhANP-mediated reduction of LPS-induced systemic inflammation and neuroinflammation. **a** Treatment schedule. Mice were intraperitoneally injected with lipopolysaccharides (LPS, 5 mg/kg). Recombinant human ANP (rhANP; 1.0 mg/kg) or 0.9% saline were intraperitoneally injected to mice at 24 h before and 10 min after LPS injection. Subdiaphragmatic vagotomy (SDV) was performed 14 days prior to LPS injection. Plasma and hippocampus were collected 24 h after LPS injection. **b** Body weight loss in each group (two-way ANOVA: rhANP: *F*_1,36_ = 7.058, *P* = 0.0117; SDV: *F*_1,36_ = 12.72, *P* = 0.0010; interaction: *F*_1,36_ = 4.923, *P* = 0.0329). The plasma levels of interleukin (IL)-6 (**c**; two-way ANOVA: rhANP: *F*_1,36_ = 13.07, *P* = 0.0009; SDV: *F*_1,36_ = 15.60, *P* = 0.0003; interaction: *F*_1,36_ = 20.51, *P* < 0.0001), IL-17A (**d**; two-way ANOVA: rhANP: *F*_1,36_ = 6.111, *P* = 0.0183; SDV: *F*_1,36_ = 7.794, *P* = 0.0083; interaction: *F*_1,36_ = 6.027, *P* = 0.0191), interferon (IFN)-γ (**e**; two-way ANOVA: rhANP: *F*_1,36_ = 6.460, *P* = 0.0155; SDV: *F*_1,36_ = 8.447, *P* = 0.0062; interaction: F_1,36_ = 7.836, *P* = 0.0082), and tumor necrosis factor (TNF)-α (**f**; two-way ANOVA: rhANP: *F*_1,36_ = 4.828, *P* = 0.0345; SDV: *F*_1,36_ = 6.217, *P* = 0.0174; interaction: *F*_1,36_ = 7.883, *P* = 0.0080). Western blot analysis of ionized calcium-binding adapter molecule 1 (iba-1) (**g**; two-way ANOVA: rhANP: *F*_1,36_ = 6.208, *P* = 0.0175; SDV: *F*_1,36_ = 4.772, *P* = 0.0355; interaction: *F*_1,36_ = 4.996, *P* = 0.0317), IL-6 (**h**; two-way ANOVA: rhANP: *F*_1,36_ = 6.286, *P* = 0.0168; SDV: *F*_1,36_ = 6.894, *P* = 0.0126; interaction: *F*_1,36_ = 8.569, *P* = 0.0059), IL-17A (**i**; two-way ANOVA: rhANP: *F*_1,36_ = 4.268, *P* = 0.0461; SDV: *F*_1,36_ = 3.363, *P* = 0.0750; interaction: *F*_1,36_ = 5.503, *P* = 0.0246), interferon (IFN)-γ (**j**; two-way ANOVA: rhANP: *F*_1,36_ = 4.706, *P* = 0.0367; SDV: *F*_1,36_ = 5.685, *P* = 0.0225; interaction: *F*_1,36_ = 3.530, *P* = 0.0684), TNF-α (**k**; two-way ANOVA: rhANP: *F*_1,36_ = 1.598, *P* = 0.2144; SDV: *F*_1,36_ = 12.72, *P* = 0.0010; interaction: *F*_1,36_ = 4.345, *P* = 0.0443), inducible nitric oxide synthase (iNOS) (**l**; two-way ANOVA: rhANP: *F*_1,36_ = 4.764, *P* = 0.0357; SDV: *F*_1,36_ = 2.933, *P* = 0.0954; interaction: *F*_1,36_ = 5.462, *P* = 0.0251) and their respective β-actin in the hippocampus. Data are shown as mean ± SEM, *n* = 10/group. ^*^P < 0.05, ^**^P < 0.01, ^***^P < 0.0001; *N.S.* not significant

**Fig. 4:**
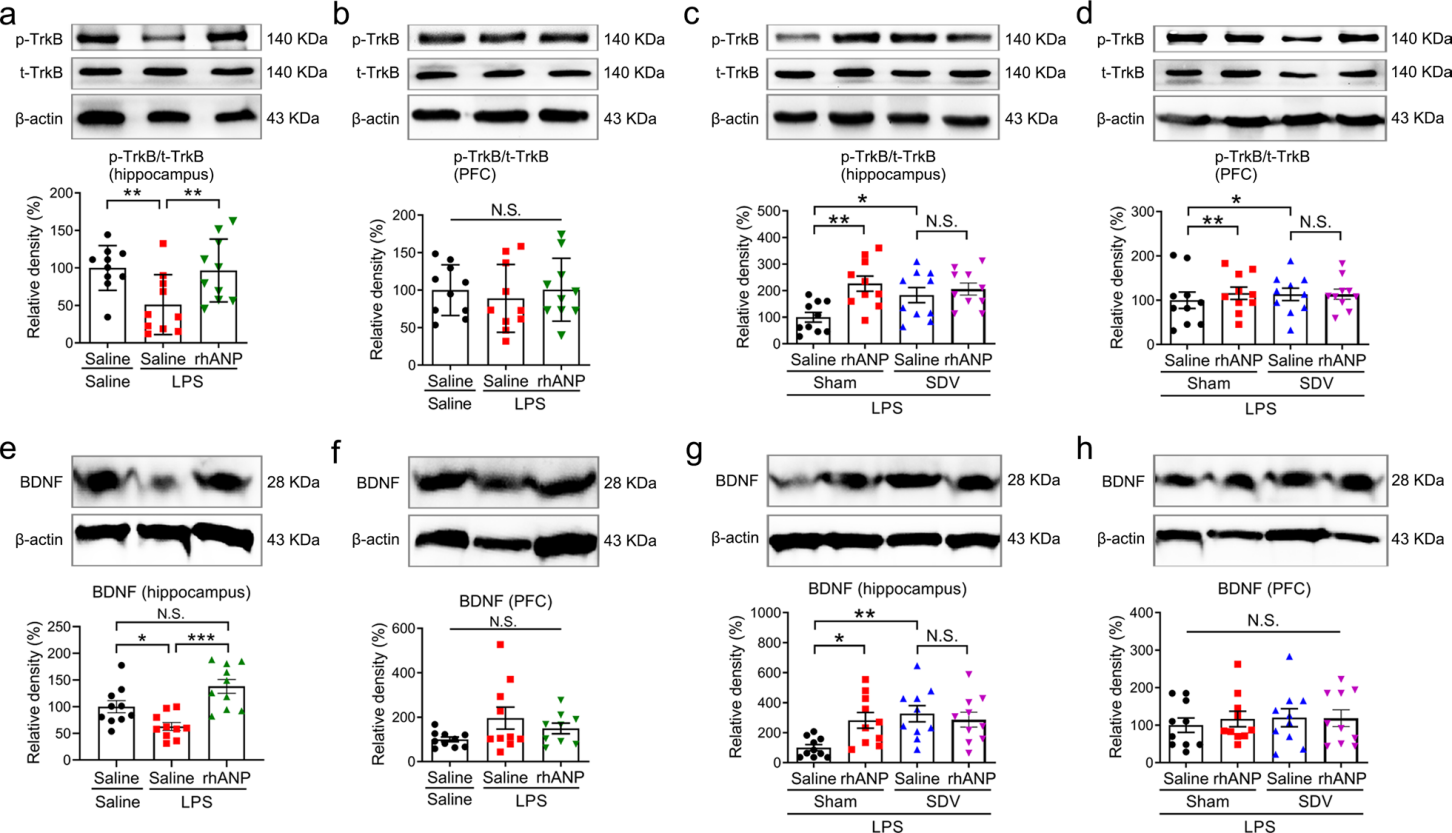
Effects of SDV on hippocampal TrkB/BDNF signaling in LPS-challenged mice treated with rhANP. **a**, **b** Western blot analysis of phosphorylated/total tyrosine kinase receptor B ratio (p-TrkB/t-total) in the hippocampus (one-way ANOVA: *F*_2,27_ = 5.245, *P* = 0.0119) and prefrontal cortex (PFC) (one-way ANOVA: *F*_2,27_ = 0.2589, *P* = 0.7738) of mice treated with recombinant human ANP (rhANP) or 0.9% saline 24 h after injection of lipopolysaccharides (LPS) or 0.9% saline. **c**, **d** Western blot analysis of p-TrkB/t-total in the hippocampus (two-way ANOVA: rhANP: *F*_1,36_ = 1.633, *P* = 0.2095; SDV: *F*_1,36_ = 9.169, *P* = 0.0045; interaction: *F*_1,36_ = 4.395, *P* = 0.0431) and PFC (two-way ANOVA: rhANP: *F*_1,36_ = 0.1409, *P* = 0.7096; SDV: *F*_1,36_ = 0.3043, *P* = 0.5846; interaction: *F*_1,36_ = 0.2733, *P* = 0.6044) of rhANP or 0.9% saline-treated endotoxemia mice pre-subjected to SDV or sham operation. **e**, **f** Western blot analysis of BDNF in the hippocampus (one-way ANOVA: *F*_2,27_ = 12.20, *P* = 0.0002) and PFC (one-way ANOVA: *F*_2,27_ = 2.218, *P* = 0.1290) of mice treated with rhANP or 0.9% saline 24 h after injection of LPS or 0.9% saline. **g**, **h** Western blot analysis of BDNF in the hippocampus (two-way ANOVA: rhANP: *F*_1,36_ = 6.305, *P* = 0.0167; SDV: *F*_1,36_ = 2.378, *P* = 0.1318; interaction: *F*_1,36_ = 5.767, *P* = 0.0216) and PFC (two-way ANOVA: rhANP: *F*_1,36_ = 0.2615, *P* = 0.6122; SDV: F_1,36_ = 0.1217, *P* = 0.7292; interaction: *F*_1,36_ = 0.1715, *P* = 0.6813) of rhANP or 0.9% saline-treated endotoxemia mice pre-subjected to SDV or sham operation. Data are shown as mean ± SEM, *n* = 10/group. ^*^P < 0.05, ^**^P < 0.01, ^***^P < 0.0001; *N.S.* not significant. *SDV* subdiaphragmatic vagotomy

**Fig. 5:**
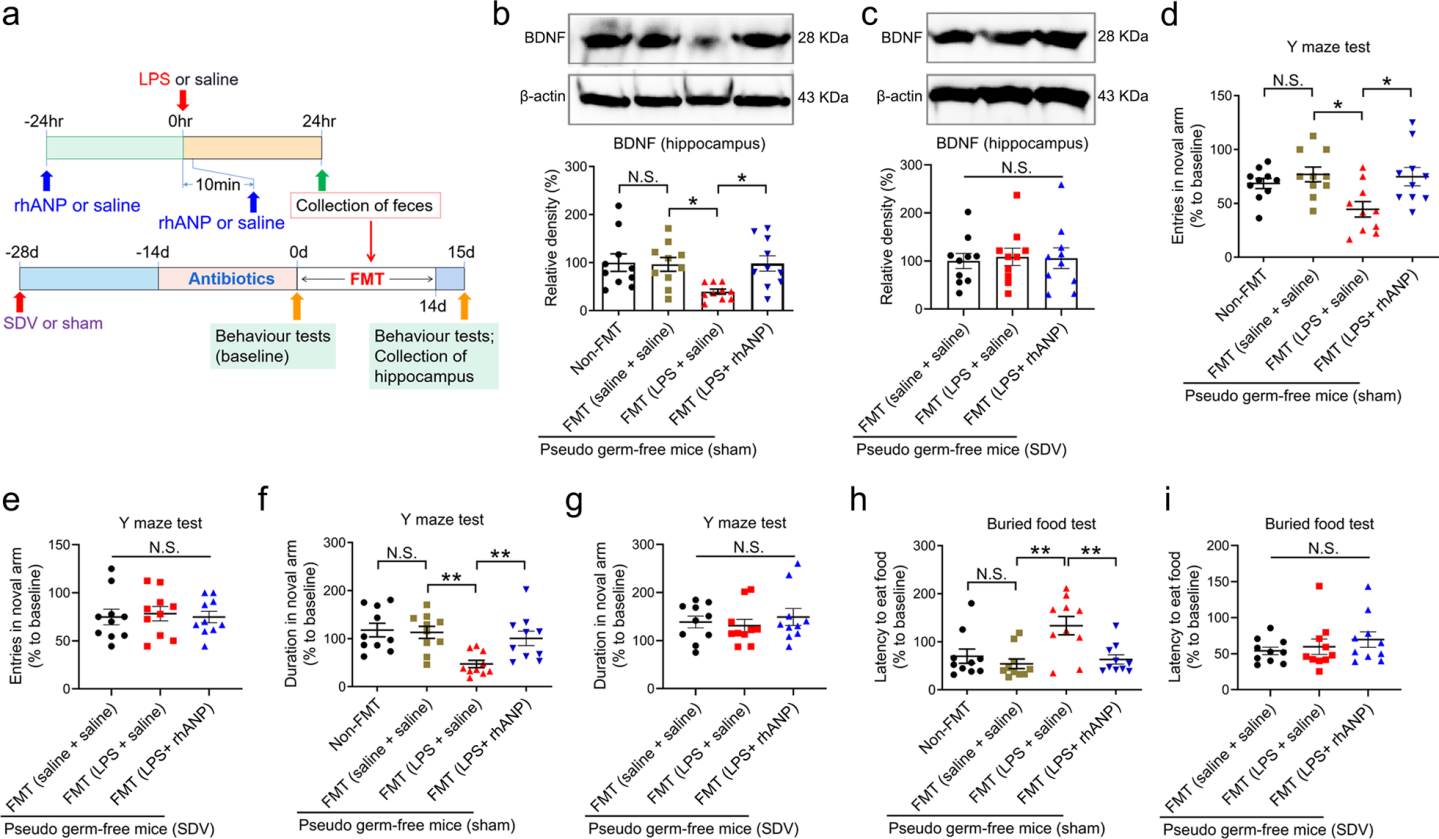
Key role of SDV in the improving effects of rhANP on disturbed gut microbiota-induced cognitive dysfunction after LPS injection. **a** Treatment schedule. The pseudo germ-free (PGF) mouse model was created by administering large doses of antibiotics to mice for 14 consecutive days. The PGF mice underwent subdiaphragmatic vagotomy (SDV) or sham operation 28 days before fecal microbiota transplantation (FMT). The PGF mice were given by gavage fecal bacteria suspension from lipopolysaccharides (LPS) or 0.9% saline-injected mice with or without recombinant human ANP (rhANP) treatment. On day 15, hippocampus was collected. Western blot analysis of BDNF in the hippocampus of PGF mice received sham operation (**b**; one-way ANOVA: *F*_3,36_ = 4.216, *P* = 0.0118) or SDV (**c**; one-way ANOVA: *F*_2,27_ = 0.05614, *P* = 0.9455). The entries in the novel arm in the Y maze test in the PGF mice subjected to sham operation (**d**; one-way ANOVA: *F*_2,27_ = 4.621, *P* = 0.0078) or SDV (**e**; one-way ANOVA: *F*_2,27_ = 0.07938, *P* = 0.9239). The duration in the novel arm in the Y maze test in the PGF mice subjected to sham operation (**f**; one-way ANOVA: *F*_3,36_ = 6.602, *P* = 0.0011) or SDV (**g**; one-way ANOVA: *F*_2,27_ = 0.3912, *P* = 0.6800). The latency to eat food in the buried food test in the PGF mice subjected to sham operation (**h**; one-way ANOVA: *F*_3,36_ = 6.701, *P* = 0.0010) or SDV (**i**; one-way ANOVA: *F*_2,27_ = 0.7501, *P* = 0.4819). Data are shown as mean ± SEM, *n* = 10/group. ^*^P < 0.05, ^**^P < 0.01; *N.S.* not significant

**Fig. 6:**
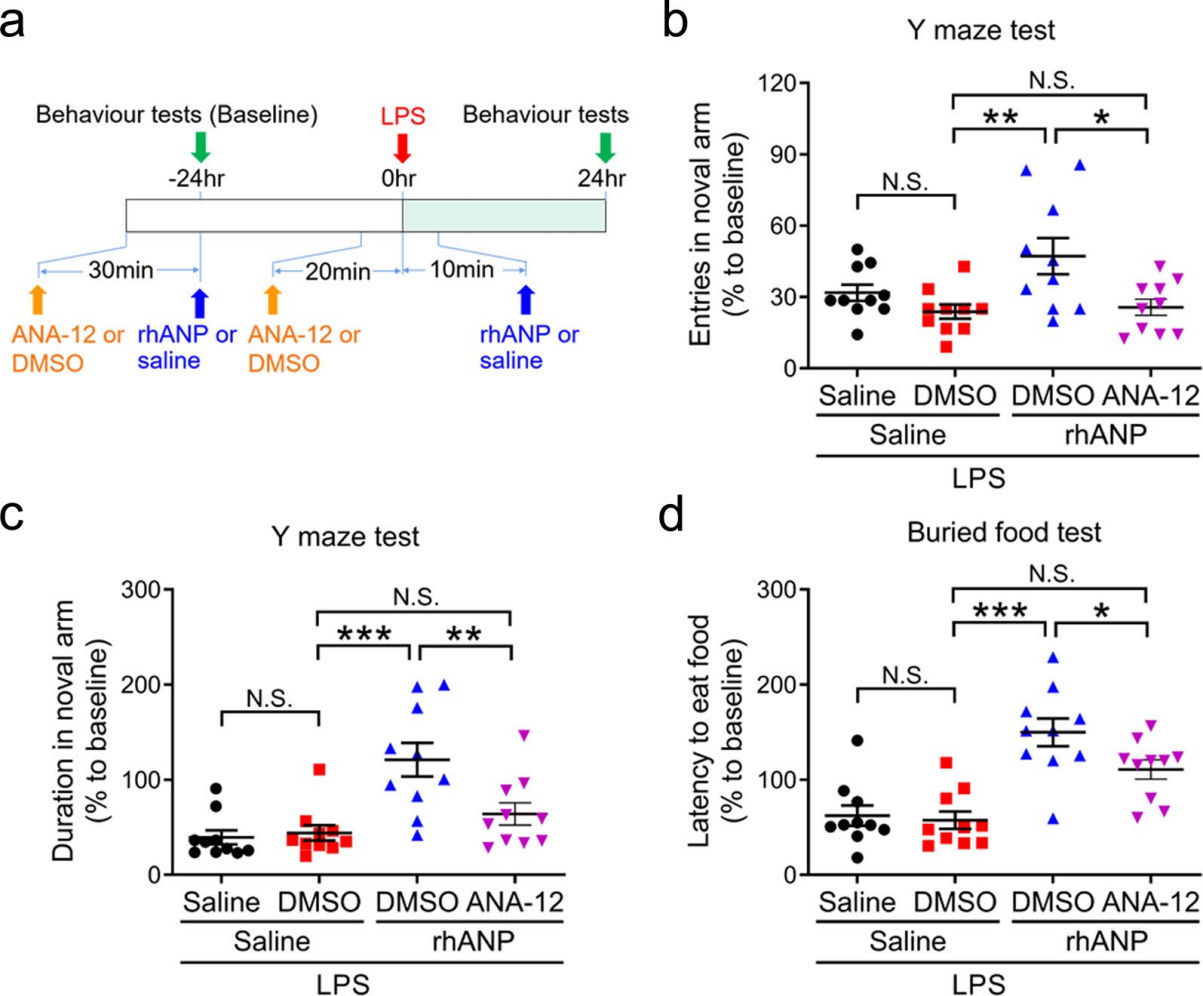
Roles of hippocampal BDNF in the improving effects of rhANP on LPS-induced cognitive dysfunction. **a** Treatment schedule. Mice were intraperitoneally injected with lipopolysaccharides (LPS, 5 mg/kg). Recombinant human ANP (rhANP; 1.0 mg/kg) or 0.9% saline (10 ml/kg) were intraperitoneally injected to mice at 24 h before and 10 min after LPS injection. ANA-12 (0.5 mg/kg) or 17% dimethylsulfoxide (DMSO) was administrated 30 min prior to rhANP treatment. Entries in the novel arm (**b**; two-way ANOVA: rhANP: *F*_1,36_ = 3.302, *P* = 0.0775; ANA-12: *F*_1,36_ = 9.635, *P* = 0.0037; interaction: *F*_1,36_ = 2.041, *P* = 0.1617) and duration in the novel arm (**c**; two-way ANOVA: rhANP: *F*_1,36_ = 18.14, *P* = 0.0001; ANA-12: *F*_1,36_ = 4.792, *P* = .0352; interaction: *F*_1,36_ = 6.668, *P* = 0.0140) in the Y maze test. **d** Latency to eat food in the buried food test (two-way ANOVA: rhANP: *F*_1,36_ = 38.53, *P* < 0.0001; ANA-12: *F*_1,36_ = 3.738, *P* = 0.0611; interaction: *F*_1,36_ = 2.262, *P* = 0.1413). Data are shown as mean ± SEM, *n* = 10/group. ^*^P < 0.05, ^**^P < 0.01, ^***^P < 0.0001; *N.S.* not significant

Data were expressed as the mean ± standard error of the mean (S.E.M). Data in Figs. 3, 4c, d and 6 were analyzed using two-way analysis of variance (ANOVA) followed by post-hoc Tukey’s multiple comparison tests. Data in Figs. 1, 2, 4a, b and 5 were analyzed using one-way ANOVA followed by post-hoc Newman–Keuls’s multiple comparison tests. Correlation was analyzed by Pearson correlation. *P* < 0.05 was considered statistically significant. Statistical analysis was performed using GraphPad prism 8 software (GraphPad Software Inc, San Diego, CA, USA).

## Results

### Effects of rhANP on spleen weight and plasma inflammatory cytokines after LPS injection

First, we investigated the effects of rhANP treated in three different protocols on the spleen weight and plasma inflammatory cytokines after LPS injection (Fig. 1a). Combined prophylactic and therapeutic treatment with rhANP (24 h before and 10 min after LPS injection) reversed LPS-induced body weight loss (Fig. 1b), increased spleen weight (Fig. 1c) and increased spleen weight/body weight ratio (Fig. 1d) at 24 h after LPS injection. A significant attenuation of LPS-induced increase in the plasma levels of IL-6, IL-17A, IFN-γ and TNF-α was observed in mice received combined prophylactic and therapeutic treatment with rhANP, as compared to that in mice treated with saline (Fig. 1e–h). Interestingly, the spleen weight was significantly positively correlated with the plasma levels of inflammatory cytokines (IL-6, IL-17A, IFN-γ or TNF-α) (Fig. 1i–l).

However, single dose administration of rhANP 24 h before or 10 min after LPS injection had no significant effects on the spleen weight, the spleen weight/body weight ratio and the plasma levels of inflammatory cytokines (IL-6, IL-17A, IFN-γ and TNF-α) (Additional file 1: Fig. S1a–g).

### Effects of rhANP on LPS-induced neuroinflammation and cognitive dysfunction

Combined prophylactic and therapeutic treatment with rhANP reversed LPS-induced increase in the protein expression of iba-1, IL-6, IL-17A, IFN-γ, TNF-α and iNOS in the hippocampus, rather than in the PFC at 24 h after LPS injection (Fig. 2b–m).

In the Y maze test, LPS-induced decrease in the number of entries and duration in the novel arm was significantly alleviated by the combined prophylactic and therapeutic treatment with rhANP at 24 h after LPS injection (Fig. 2n, o). Combined prophylactic and therapeutic treatment with rhANP also significantly attenuated LPS-induced increase in the latency of mice to eat the food in the buried food test at 24 h after LPS injection (Fig. 2p). However, single dose administration of rhANP 24 h before or 10 min after LPS injection had no significant effects on the latency to eat the food at 24 h after LPS injection (Additional file 1: Fig. S1h).

### Roles of SDV in rhANP-mediated reduction of LPS-induced systemic inflammation and neuroinflammation

Next, we studied the mechanisms underlying the protective effects of rhANP on the systemic LPS-induced systemic inflammation, neuroinflammation and cognitive dysfunction. SDV was performed 14 days before systemic LPS injection (Fig. 3a). We found that LPS-induced increase in the body weight loss, plasma levels of inflammatory cytokines (IL-6, IL-17A, IFN-γ and TNF-α; Fig. 3b–f) and protein expression of hippocampal inflammatory mediators (iba-1, IL-6, IL-17A, IFN-γ, TNF-α and iNOS) was significantly attenuated by SDV (Fig. 3g–l). However, combined prophylactic and therapeutic treatment with rhANP could not further decrease the body weight loss, plasma levels of inflammatory cytokines and protein expression of hippocampal inflammatory mediators in LPS-challenged mice received SDV (Fig. 3b–l), suggesting that rhANP might exert its anti-inflammatory effects through subdiaphragmatic vagus nerve.

### Effects of SDV on hippocampal TrkB/BDNF signaling in LPS-challenged mice treated with rhANP

LPS-challenged mice treated twice with rhANP had significantly higher expression of p-TrkB (Fig. 4a) and BDNF in the hippocampus (Fig. 4e), rather than in the PFC (Fig. 4b, f), as compared to LPS-challenged mice treated twice with 0.9% saline. SDV significantly increased the expression of p-TrkB (Fig. 4c) and BDNF (Fig. 4g) in the hippocampus, but not in the PFC (Fig. 4d, h), compared to sham operation in LPS-challenged mice. However, combined prophylactic and therapeutic treatment with rhANP could not further increase the hippocampal levels of p-TrkB or BDNF in LPS-challenged mice received SDV (Fig. 4c, g).

### Essential role of subdiaphragmatic vagus nerve in the improving effects of rhANP on disturbed gut microbiota-induced cognitive dysfunction after LPS injection

It has been shown that subdiaphragmatic vagus nerve plays important regulatory roles in gut microbiota–brain axis after endotoxemia or sepsis [33, 34]. In our study, PGF mouse model was created by administering large doses of antibiotics to mice for 14 consecutive days, and then FMT was performed by oral gavage in the PGF mice using the fecal bacteria suspension obtained from 0.9% saline or LPS-challenged mice with or without rhANP treatment (Fig. 5a). We found that PGF mice transplanted with fecal bacteria suspension from LPS-challenged mice had significantly decreased hippocampal BDNF expression (Fig. 5b), decreased number of entries and duration in the novel arm in the Y maze test (Fig. 5d, f), and increased latency to eat the food in the buried food test (Fig. 5h) than PGF mice transplanted with fecal bacteria suspension from 0.9% saline-treated mice or LPS-challenged mice treated with rhANP. In PGF mice pre-received SDV, transplantation with fecal bacteria suspension from LPS-challenged mice with or without rhANP treatment could not significantly decrease the hippocampal BDNF expression (Fig. 5c), decrease the number of entries and duration in the novel arm in the Y maze test (Fig. 5e, g), and increase the latency to eat the food in the buried food test (Fig. 5i), indicating an essential role of subdiaphragmatic vagus nerve in disturbed gut microbiota-induced cognitive dysfunction after LPS injection.

### Roles of hippocampal BDNF in the improving effects of rhANP on LPS-induced cognitive dysfunction

In view of the important role of hippocampal BDNF in synaptic plasticity, learning and memory function [38], we sought to examine the role of BDNF in the improving effects of rhANP on LPS-induced cognitive dysfunction. ANA-12 was administrated to LPS-challenged mice 30 min prior to rhANP treatment to block TrkB/BDNF signaling (Fig.3 and Additional file 1: Fig. S2). We found that the increased number of entries and duration in the novel arm in the Y maze test (Fig. 6b, c) and the decreased latency to eat the food in the buried food test (Fig. 6d) induced by combined prophylactic and therapeutic treatment with rhANP in LPS-challenged mice were significantly weakened by treatment with ANA-12.

## Discussion

In the present study, our results showed that combined prophylactic and therapeutic treatment with rhANP could alleviate systemic inflammation, neuroinflammation and cognitive dysfunction induced by intraperitoneal injection of LPS in mice, as indicated by the decreased plasma proinflammatory cytokines, decreased protein levels of inflammatory mediators in the hippocampus, increased number of entries and duration in the novel arm, and decreased latency to eat the food in the buried food test. Moreover, the mechanism of its cognitive improving effects could be associated with its ability in regulating hippocampus TrkB/BDNF signaling by modulating gut microbiota composition and then by conduction of subdiaphragmatic vagus nerve.

ANP has been shown to be released mainly from heart atria [16], which could exert immunomodulatory capacity and anti-inflammatory properties, including inhibiting the expression of proinflammatory mediators (TNF-α, IL-1, monocyte chemoattractant protein 1, nitric oxide, cyclooxygenase-2, etc.) and adhesion molecules (vascular cell adhesion molecules, intercellular cell adhesion molecule-1, E-selectin, etc.) [17, 28–31], suppressing LPS and TNF-α-induced increased permeability of endothelial cells [18], and alleviating post-septic intestinal injury [25]. However, the molecular mechanisms by which ANP modulates immune responses/immune cell activation are unclear. In our present study, combined prophylactic and therapeutic treatment with rhANP attenuated systemic LPS-triggered systemic inflammation, neuroinflammation and cognitive dysfunction, which might act through altered gut microbiota composition. PGF mice transplanted with fecal bacteria suspension from rhANP-treated endotoxemia mice had less severity of cognitive dysfunction than PGF mice transplanted with fecal bacteria suspension from 0.9% saline-treated endotoxemia mice, indicating an essential role of gut microbiota in rhANP-mediated attenuation of cognitive dysfunction after endotoxemia. However, we did not perform 16S rRNA high-throughput sequencing to examine the roles and mechanisms of rhANP treatment in the alteration of gut microbiota composition after LPS-induced endotoxemia, which is certainly a limitation of our present study. Further researches are needed to clarify the effects of rhANP treatment on the families, genera, and species of gut microbiota after systemic LPS-induced endotoxemia in the future. It has been shown that natriuretic peptide receptor A (NPR-A), a major receptor for ANP, is highly expressed in small intestinal epithelial cells [35]. Therefore, we speculate that circulatory ANP secreted from heart may improve disturbed gut microbiota through its binding to NPR-A in the small intestine, which needs to be further studied in the future. However, the mechanisms of how the heart is stimulated to secret ANP and how the ANP crosses the gut barriers and binds to the NPR-A in the small intestine remain unclear and is well worthy of further study. Studies have demonstrated that systemic LPS injection could directly disrupt the integrity and function of BBB [4–7], and activated microglias play important roles in LPS-induced neuroinflammation [11, 39]. Therefore, whether rhANP could directly act on activated microglia to exert neuroprotective effects against LPS-induced endotoxemia remains unclear, which is a limitation of our study and needs further study in the future.

In our present study, only combined prophylactic and therapeutic treatment with rhANP could attenuate systemic inflammation, neuroinflammation and cognitive dysfunction, which is consistent with our recent results that (*R*)-Ketamine could exert its protective effects only when used prophylactically and therapeutically in a small dose of LPS-induced mouse model of depression or cecal ligation and puncture-induced severe sepsis [37, 40]. Our findings highlight the importance of the pre-administration of anti-inflammatory drugs. Prophylactic treatment is critical for patients surviving endotoxemia or sepsis who are susceptible to secondary infections due to sustained immune suppression, or for immunocompromised patients who are prone to develop endotoxemia or sepsis.

BDNF expressed in hippocampus and cerebral cortex plays important roles in the synaptic plasticity, neuronal survival and memory formation via binding and activating its high affinity receptor TrkB [41]. The hippocampal-specific deletion of the BDNF gene by a lentivirus expressing Cre recombinase impairs spatial learning and extinction of aversive memories [42]. Our data found that systemic LPS challenge decreased the p-TrkB and BDNF levels in the hippocampus, which is in line with a previous study [43]. Combined prophylactic and therapeutic treatment with rhANP-mediated attenuation of neuroinflammation and cognitive impairment was accompanied by a up-regulation of p-TrkB and BDNF in the hippocampus. Blocking hippocampal TrkB/BDNF signaling with ANA-12 abrogated the improving effects of rhANP on LPS-induced cognitive dysfunction. The PFC has been shown to control cognitive and emotional behaviors [44, 45]. The connections between the hippocampus and PFC are critical for cognition, emotion and memory, PFC activity plays important roles in the maintenance of mnemonic and spatial representations in hippocampus or posterior parietal cortex [46–48]. However, no significant effects were observed in the expression of inflammatory mediators as wells as p-TrkB and BDNF in the PFC after combined prophylactic and therapeutic treatment with rhANP in LPS-induced endotoxemia, indicating rhANP could exert its anti-neuroinflammatory and cognition-improving effects specifically via hippocampal TrkB/BDNF signaling pathway.

In our previous studies, we have shown that subdiaphragmatic vagus nerve plays important regulatory roles in gut microbiota–brain axis after endotoxemia or sepsis [33, 34]. In mice with LPS (5 mg/kg)-induced endotoxemia, the increased translocation of gut microbial components and higher mortality caused by Mucin 2 deficient (*Muc2*^*−/−*^) could be significantly improved by the pre-administration of broad-spectrum antibiotic cocktail before endotoxemia. *Muc2*^*−/−*^ no longer increases mortality after LPS-induced endotoxemia in germ-free (GF) mice [49], indicating an essential role of gut microbiota in pathogenic factors-aggravated systemic inflammatory injury after endotoxemia. Ingestion of beneficial bacteria (*L. rhamnosus*, *Bifidobacterium longum* NCC3001) alleviates stress-induced anxiety and depression-like behaviors, which is abolished by SDV [50–52]. A disturbance of gut microbiota is evident after sepsis or systemic LPS-induced endotoxemia [33, 53]. We and others have previously demonstrated that SDV could block the depression-like phenotype induced by systemic LPS challenge in rodents [33]. The alterations in the gut microbiota induced by red light exposure after a large dose of systemic LPS-induced sepsis impair cognitive function and induce anxiety-like behavior through subdiaphragmatic vagus nerve [34]. The above research results suggest that subdiaphragmatic vagus nerve could mediate the communication between the brain and the gut microbiota to exert beneficial or detrimental effects depending on the predominance of probiotic or pathogenic microorganisms present in the intestinal flora. After SDV, systemic LPS challenge with or without treatment with anti-inflammatory therapeutic agents could not affect systemic inflammation, neuroinflammation or neuropsychiatric disorders, regardless of whether the gut microbiota composition has changed markedly. Therefore, the integrity of subdiaphragmatic vagus nerve might play a critical role in the anti-neuroinflammatory and cognition-improving effects of therapeutic agents through modulating gut microbiota composition after systemic LPS-induced endotoxemia or sepsis. In our present study, we found that SDV abrogated gut microbiota alteration-induced neuroinflammation and cognitive dysfunction in endotoxemia mice after systemic LPS challenge, regardless of whether rhANP treatment was given, indicating that rhANP might attenuate LPS-triggered systemic inflammation, neuroinflammation and cognitive dysfunction by modulating gut microbiota composition and then by the conduction of subdiaphragmatic vagus nerve. However, the mechanisms by which altered gut microbiota regulates the hippocampal TrkB/BDNF signaling after the conduction of subdiaphragmatic vagus nerve remain unclear and need further study.

## Conclusions

In summary, our study revealed that rhANP could alleviate LPS-induced systemic inflammation, neuroinflammation and cognitive dysfunction via activating hippocampal TrkB/BDNF signaling in mice, the mechanism of which might be through subdiaphragmatic vagus nerve-mediated gut microbiota–brain axis.

## Supplementary Information

**Additional file 1: Fig. S1.** Effects of prophylactic or therapeutic use of rhANP on spleen weight, plasma inflammatory cytokines and cognitive function after LPS-triggered endotoxemia. a Treatment schedule. Mice were intraperitoneally injected with lipopolysaccharides (LPS, 5 mg/kg) or 0.9% saline (10 ml/kg). Recombinant human ANP (rhANP; 1.0 mg/kg) or 0.9% saline (10 ml/kg) were intraperitoneally injected to mice at 24 h before or 10 min after LPS injection. Spleen and plasma were collected 24 h after injection of LPS or 0.9% saline. b Spleen weight (one-way ANOVA: F_3,36_ = 7.004, *P* = 0.0008). c The ratio of spleen weight/body weight (one-way ANOVA: F_3,36_ = 8.996, *P* = 0.0001). Plasma levels of interleukin (IL)-6 (d; F_3,36_ = 13.93, *P* < 0.0001), IL-17A (e; one-way ANOVA: F_3,36_ = 6.195, *P* = 0.0017), interferon (IFN)-γ (f; one-way ANOVA: F_3,36_ = 3.903, *P* = 0.0164) and tumor necrosis factor (TNF)-α (g; one-way ANOVA: F_3,36_ = 8.209, *P* < 0.0001) in each group. (h) The latency of mice to eat food in the buried food test (one-way ANOVA: F_3,36_ = 8.162, *P* = 0.0003). Data are shown as mean ± SEM, n = 10/group. ^*^P **<** 0.05, ^**^P < 0.01, ^***^P < 0.0001; N.S. not significant. **Fig. S2.** Effects of ANA-12 on the activation of TrkB/BDNF signaling in the hippocampus. a Treatment schedule. Mice were intraperitoneally injected with lipopolysaccharides (LPS, 5 mg/kg). Recombinant human ANP (rhANP; 1.0 mg/kg) was intraperitoneally injected to mice at 24 h before and 10 min after LPS injection. ANA-12 (0.5mg/kg) or 17% dimethylsulfoxide (DMSO) was administrated 30 min prior to rhANP treatment. Western blot analysis of phosphorylated/total tyrosine kinase receptor B ratio (p-TrkB/t-total; b), brain-derived neurotrophic factor (BDNF; c) in the hippocampus 24 h after injection of LPS. Data are shown as mean ± SEM, n = 10/group. ^*^P < 0.05, ^**^P < 0.01.

## Data Availability

The data sets used and/or analyzed during the current study are available from the corresponding author on reasonable request.

## Abbreviations

ANP: Atrial natriuretic peptide
RhANP: Recombinant human ANP
LPS: Lipopolysaccharide
SDV: Subdiaphragmatic vagotomy
FMT: Fecal microbiota transplantation
iba-1: Ionized calcium-binding adaptor molecule-1
iNOS: Inducible nitric oxide synthase
BBB: Blood–brain barrier
PGF: Pseudo germ-free
PBS: Phosphate-buffered saline
PFC: Prefrontal cortex
ELISA: Enzyme-linked immunosorbent assay
IL-6: Interleukin-6
IFN-γ: Interferon-γ
BDNF: Brain-derived neurotrophic factor
TrkB: Tyrosine kinase receptor B
NPR-A: Natriuretic peptide receptor A
