# Supplementary material for: RhANP attenuates endotoxin-derived cognitive dysfunction through subdiaphragmatic vagus nerve-mediated gut microbiota–brain axis
Source: J Neuroinflammation. 2021 Dec 23;18:300. doi: 10.1186/s12974-021-02356-z (PMC8697447; doi:10.1186/s12974-021-02356-z)
Supplement: Supplementary file 1 — Additional file 1: Fig. S1. Effects of prophylactic or therapeutic use of rhANP on spleen weight, plasma inflammatory cytokines and cognitive function after LPS-triggered endotoxemia. a Treatment schedule. Mice were intraperitoneally injected with lipopolysaccharides (LPS, 5 mg/kg) or 0.9% saline (10 ml/kg). Recombinant human ANP (rhANP; 1.0 mg/kg) or 0.9% saline (10 ml/kg) were intraperitoneally injected to mice at 24 h before or 10 min after LPS injection. Spleen and plasma were collected 24 h after injection of LPS or 0.9% saline. b Spleen weight (one-way ANOVA: F3,36 = 7.004, P = 0.0008). c The ratio of spleen weight/body weight (one-way ANOVA: F3,36 = 8.996, P = 0.0001). Plasma levels of interleukin (IL)-6 (d; F3,36 = 13.93, P < 0.0001), IL-17A (e; one-way ANOVA: F3,36 = 6.195, P = 0.0017), interferon (IFN)-γ (f; one-way ANOVA: F3,36 = 3.903, P = 0.0164) and tumor necrosis factor (TNF)-α (g; one-way ANOVA: F3,36 = 8.209, P < 0.0001) in each group. (h) The latency of mice to eat food in the buried food test (one-way ANOVA: F3,36 = 8.162, P = 0.0003). Data are shown as mean ± SEM, n = 10/group. *P < 0.05, **P < 0.01, ***P < 0.0001; N.S. not significant. Fig. S2. Effects of ANA-12 on the activation of TrkB/BDNF signaling in the hippocampus. a Treatment schedule. Mice were intraperitoneally injected with lipopolysaccharides (LPS, 5 mg/kg). Recombinant human ANP (rhANP; 1.0 mg/kg) was intraperitoneally injected to mice at 24 h before and 10 min after LPS injection. ANA-12 (0.5mg/kg) or 17% dimethylsulfoxide (DMSO) was administrated 30 min prior to rhANP treatment. Western blot analysis of phosphorylated/total tyrosine kinase receptor B ratio (p-TrkB/t-total; b), brain-derived neurotrophic factor (BDNF; c) in the hippocampus 24 h after injection of LPS. Data are shown as mean ± SEM, n = 10/group. *P < 0.05, **P < 0.01. [file 12974_2021_2356_MOESM1_ESM.docx]

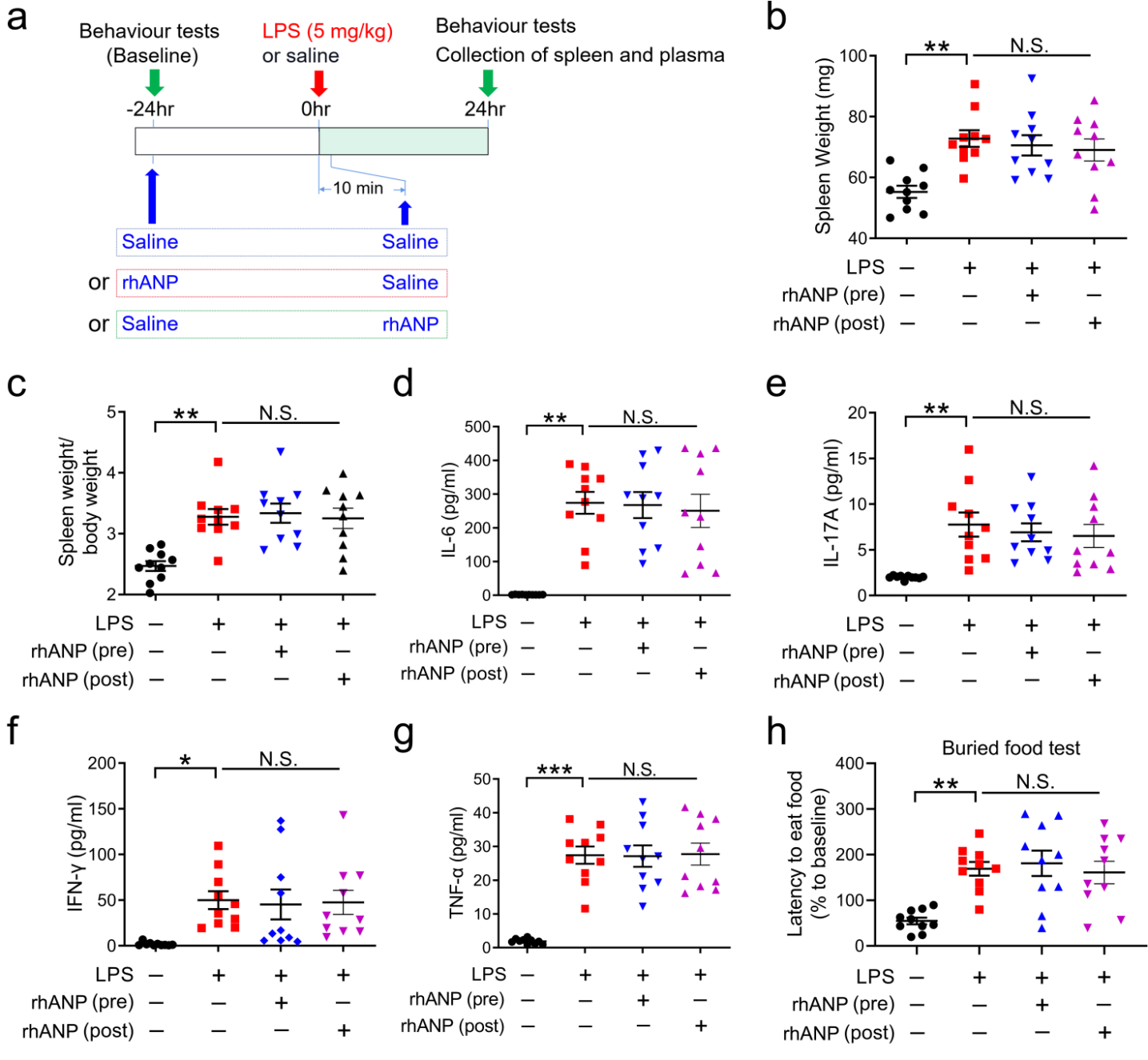


**Fig. S1**

**Effects of prophylactic or therapeutic use of rhANP on spleen weight, plasma inflammatory cytokines and cognitive function after LPS-triggered endotoxemia. a** Treatment schedule. Mice were intraperitoneally injected with lipopolysaccharides (LPS, 5 mg/kg) or 0.9% saline (10 ml/kg). Recombinant human ANP (rhANP; 1.0 mg/kg) or 0.9% saline (10 ml/kg) were intraperitoneally injected to mice at 24 hours before or 10 minutes after LPS injection. Spleen and plasma were collected 24 hours after injection of LPS or 0.9% saline. **b** Spleen weight (one way ANOVA: F_3,36_ = 7.004, *P* = 0.0008). **c** The ratio of spleen weight/body weight (one way ANOVA: F_3,36_ = 8.996, *P* = 0.0001). Plasma levels of interleukin (IL)-6 (**d**; F_3,36_ = 13.93, *P* < 0.0001), IL-17A (**e**; one way ANOVA: F_3,36_ = 6.195, *P* = 0.0017), interferon (IFN)-γ (**f**; one way ANOVA: F_3,36_ = 3.903, *P* = 0.0164) and tumor necrosis factor (TNF)-α (**g**; one way ANOVA: F_3,36_ = 8.209, *P* < 0.0001) in each group. (**h**) The latency of mice to eat food in the buried food test (one way ANOVA: F_3,36_ = 8.162, *P* = 0.0003). Data are shown as mean ± SEM, n = 10/group. ^*^*P* < 0.05, ^**^*P* < 0.01, ^***^*P* < 0.0001; N.S. not significant.


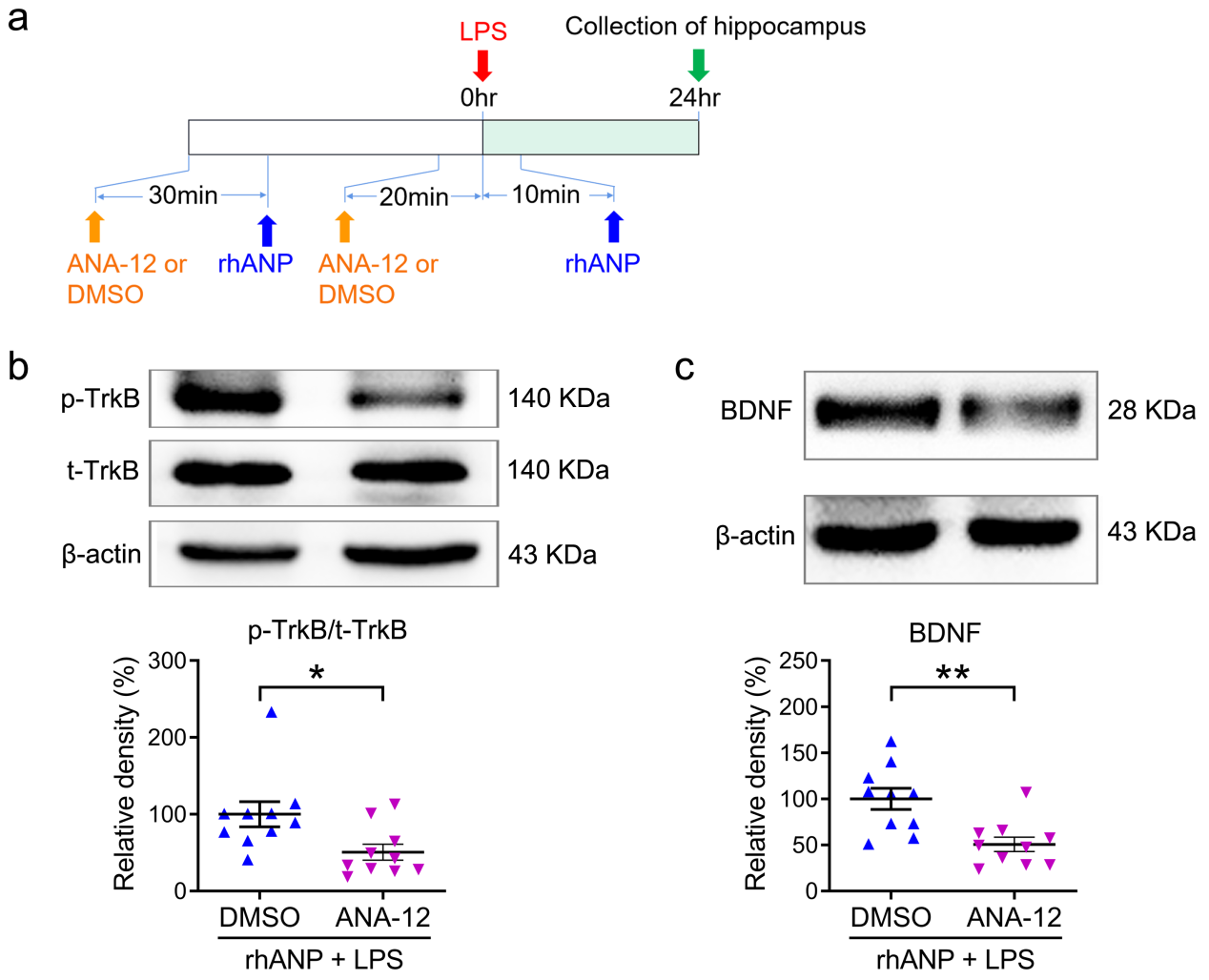


**Fig. S2**

**Effects of ANA-12 on the activation of TrkB/BDNF signaling in the hippocampus**. **a** Treatment schedule. Mice were intraperitoneally injected with lipopolysaccharides (LPS, 5 mg/kg). Recombinant human ANP (rhANP; 1.0 mg/kg) was intraperitoneally injected to mice at 24 hours before and 10 minutes after LPS injection. ANA-12 (0.5mg/kg) or 17% dimethylsulfoxide (DMSO) was administrated 30 minutes prior to rhANP treatment. Western blot analysis of phosphorylated/total tyrosine kinase receptor B ratio (p-TrkB/t-total; **b**), brain-derived neurotrophic factor (BDNF; **c**) in the hippocampus 24 hours after injection of LPS. Data are shown as mean ± SEM, n = 10/group. ^*^*P* < 0.05, ^**^*P* < 0.01.
